# A Systematic Review of Paired Associative Stimulation (PAS) to Modulate Lower Limb Corticomotor Excitability: Implications for Stimulation Parameter Selection and Experimental Design

**DOI:** 10.3389/fnins.2019.00895

**Published:** 2019-08-27

**Authors:** Gemma Alder, Nada Signal, Sharon Olsen, Denise Taylor

**Affiliations:** Health and Rehabilitation Research Institute, Auckland University of Technology, Auckland, New Zealand

**Keywords:** paired associative stimulation, transcranial magnetic stimulation, cortical excitability, neuronal plasticity (MeSH), STDP, primary motor cortex, rehabilitation (MeSH), stroke (MeSH)

## Abstract

Non-invasive neuromodulatory interventions have the potential to influence neural plasticity and augment motor rehabilitation in people with stroke. Paired associative stimulation (PAS) involves the repeated pairing of single pulses of electrical stimulation to a peripheral nerve and single pulses of transcranial magnetic stimulation over the contralateral primary motor cortex. Efficacy of PAS in the lower limb of healthy and stroke populations has not been systematically appraised. Optimal protocols including stimulation parameter settings have yet to be determined. This systematic review (a) examines the efficacy of PAS on lower limb corticomotor excitability in healthy and stroke populations and (b) evaluates the stimulation parameters employed. Five databases were searched for randomized, non-randomized, and pre-post experimental studies evaluating lower limb PAS in healthy and stroke populations. Two independent reviewers identified eligible studies and assessed methodological quality using a modified Downs and Blacks Tool and the TMS Checklist. Intervention stimulation parameters and TMS measurement details were also extracted and compared. Twelve articles, comprising 24 experiments, met the inclusion criteria. Four articles evaluated PAS in people with stroke. Following a single session of PAS, 21 experiments reported modulation of corticomotor excitability, lasting up to 60 min; however, the research lacked methodological rigor. Intervention stimulation parameters were highly variable across experiments, and whilst these appeared to influence efficacy, variations in the intervention and outcome assessment methods hindered the ability to draw conclusions about optimal parameters. Lower limb PAS research requires further investigation before considering its translation into clinical practice. Eight key recommendations serve as guide for enhancing future research in the field.

## Introduction

Non-invasive neuromodulatory interventions such as repetitive transcranial magnetic stimulation (rTMS), transcranial direct current stimulation (tDCS), and paired associative stimulation (PAS) have emerged in recent years in response to an increased understanding of neural plasticity as an adaptive process (Vallence and Ridding, 2014). These interventions modulate the excitability of cortical and spinal neurons to enhance neural connectivity and learning (Wessel et al., 2015). Non-invasive neuromodulatory interventions are increasingly being investigated as methods to promote neural plasticity and functional motor recovery following acquired brain injury such as stroke (Hummel and Cohen, 2006). This review focuses on lower limb PAS a neuromodulatory intervention that uses temporally paired transcranial magnetic stimulation (TMS) and peripheral electrical stimulation to modulate neural plasticity and is therefore distinct from other neuromodulatory interventions in its delivery method and likely mechanism of action. To date the evidence for this technique has not been systematically reviewed.

PAS typically involves the repeated pairing of single pulses of electrical stimulation to a peripheral nerve with single pulses of transcranial magnetic stimulation (TMS) over the corresponding primary motor cortex (M1) (Stefan et al., 2000, 2002). PAS results in a rapid change in corticomotor excitability (CME) of the corticospinal pathway to the target muscle. This change in CME is believed to be dependent on the temporal pairing of the two stimuli in the M1, which can be altered by manipulating the interstimulus interval (ISI). When the peripheral afferent stimulus arrives in the M1 in synchrony with, or just prior to, the TMS stimulus, there is an increase in excitability of the targeted corticospinal pathway (facilitatory PAS); whereas, when the peripheral afferent stimulus arrives after the TMS stimulus, corticomotor inhibition is observed (inhibitory PAS; Wolters et al., 2003). PAS has been likened to the cellular learning process, spike-timing-dependent neural plasticity (STDP) observed in animals (Bi and Poo, 1998; Zhang et al., 1998; Jacob et al., 2007), and humans *in vitro* (Magee and Johnston, 1997; Markram et al., 1997), where changes in the order and timing of pre- and post-synaptic stimuli determine whether there is an increase or decrease in synaptic efficacy (Fröhlich, 2016). Whilst stimulation parameters vary between studies, to date, optimization of PAS has primarily focused on the ISI; where the ISI is based on either the estimated conduction time from the peripheral nerve to the M1, or is individualized to the sensory evoked potential (SEP) or motor evoked potential (MEP) latency (Wolters et al., 2003; Stinear and Hornby, 2005; Mrachacz-Kersting et al., 2007; Roy et al., 2007; Ilić et al., 2011; Kumpulainen et al., 2015). Less is known about the influence of other PAS parameters, such as stimulus intensity, stimulation frequency, or the optimal contraction state of the target muscle during stimulation.

PAS has been extensively investigated in the upper limb (Carson and Kennedy, 2013; Wischnewski and Schutter, 2016; Suppa et al., 2017); however there has been less focus on its effects in the lower limb. The neural mechanisms for the control of the lower limb differ from those in the upper limb and such differences in cortical and spinal circuity may alter the response to PAS (Brouwer and Ashby, 1992; Aymard et al., 2000; Dalpozzo et al., 2002; Volz et al., 2015; Charalambous et al., 2016). For PAS to be considered as a therapeutic tool, particularly if walking is a primary focus of rehabilitation after stroke (Dobkin, 2004; Jette et al., 2005; Latham et al., 2005; Vincent et al., 2007), its efficacy in the lower limb must be evaluated. In addition, the influence of various stimulation parameters on intervention efficacy must be considered.

Two narrative reviews have proposed that lower limb PAS is efficacious in healthy people and shows promise for promoting motor recovery in people with stroke (Carson and Kennedy, 2013; Suppa et al., 2017), but to our knowledge there has been no systematic review to ascertain its effect on the lower limb in these populations. A systematic approach to evaluating the research evidence is essential when considering the translation of neuroscience research to clinical populations and clinical practice. In particular the rigor of the experimental method is paramount. Therefore, the primary aim of this systematic review was to determine the efficacy of PAS on lower limb CME in healthy and stroke populations, whilst explicitly critiquing the methodological quality of the research and the stimulation parameters utilized during PAS interventions.

## Method

A systematic review of the literature was undertaken using the methodology defined by the Preferred Reporting Items for Systematic Review and Meta-Analysis Protocols (PRISMA-P) statement (Moher et al., 2015).

### Search Strategy

A literature search was carried out using the following electronic databases: EBSCO (CINAHL plus, MEDLINE, SPORTDiscus), Scopus, Web of Science (Neurosciences, Engineering Biomedical, and Rehabilitation) and Ovid (AMED). Search terms are presented in Table 1. Search terms were entered using truncation and wild card characters, and abbreviations were also included in the search. Additional citations were identified by hand searching reference lists of relevant studies, and through electronic searches of relevant author names.

**Table 1:** Search terms.

	**Healthy population**	**Stroke population**
Participants		Stroke OR CVA OR cerebrovascular accident OR hemipleg* OR pares*
Intervention	pair* associat* stim* OR PAS OR dual stim*	
Outcomes	cortical excit*OR cortical motor excit* OR corticomotor excit* OR corticospinal excit* OR long term potentiation OR LTP OR LTP like OR spike timing dependent plast* OR STPD OR synapse specific assoc* plast*OR motor evoke* potential* OR MEP OR transcranial magnetic stimulation OR TMS	

A second search was performed including search terms related to stroke, to ensure relevant literature related to the stroke population had not been missed. Two independent reviewers (GA, NS) screened titles and abstracts, and where necessary, the full-text publication was reviewed for eligibility according to the criteria in Table 2. If there was any uncertainty about inclusion, a third reviewer (DT) was consulted until a consensus was reached. The independent reviewers were not blinded to the study authors, institutes or journal titles. The literature search was last performed on the 10th of March 2019.

**Table 2:** Inclusion and exclusion criteria for selected studies.

	**Inclusion**	**Exclusion**
Participants	Aged over 18 years Either healthy or with a primary diagnosis of stroke with motor deficit in the affected lower limb (there were no restrictions on the type of stroke, lesion location, time since stroke, or severity of lower limb motor deficit).	Animal studies Participants experiencing neurological disorders other than stroke.
Intervention	Traditional lower limb PAS interventions, defined as the repeated pairing of pulses of peripheral electrical stimulation to a peripheral nerve with pulses of TMS over the contralateral primary motor cortex to induce excitation or inhibition.	PAS interventions targeting the spinal region, upper limb, both cerebral hemispheres, brain areas outside the primary motor cortex, or PAS combined with other neuromodulatory interventions.
Comparison	Either no intervention, sham intervention, or traditional lower limb PAS with different stimulation parameters.	
Outcomes	Corticomotor excitability as measured by motor evoked potential (MEP) amplitude, area, or stimulus response curves, induced with either single or paired pulse TMS to the primary motor cortex. Randomized, non-randomized, and pre-post experimental studies.	
Trial design	Original primary data collected pre- and post-intervention to establish the net effect of PAS.	Case reports
Data reported	Full-text peer-reviewed journals, in English, between January 2000 and March 2019.	
Type of publications		Review articles, conference proceedings, articles containing anecdotal descriptions, and expert opinions.

### Data Extraction

Details of the study design, sample size, participant characteristics, target muscle, stimulation parameters (ISI, number of pairings, frequency, intensity, pulse width, waveform, duration, dose, electrode location, resting, or active muscle state), outcome measurement technique (single pulse TMS, as a measure of CME, or paired pulse TMS, as a measure of intracortical excitability), and study findings, were extracted from the included studies.

### Quality Assessment

Methodological quality was assessed by two independent reviewers (GA, NS) using the modified Downs and Blacks quality checklist (Downs and Black, 1998) and the TMS Quality Checklist (Chipchase et al., 2012). Any disagreement was discussed with a third reviewer (DT) until consensus was achieved. All reviewers have experience in the application of TMS and experimental neurophysiological research methods in both healthy and stroke populations (Lewis et al., 2014; Jochumsen et al., 2016, 2019; Olsen et al., 2018).

The modified Downs and Blacks quality checklist evaluates the methodological quality of randomized and non-randomized studies and is commonly used in rehabilitation systematic reviews (Eng et al., 2007; Bastani and Jaberzadeh, 2012; Aubut et al., 2013; Uiga et al., 2015). It consists of 27 questions related to reporting, external validity, internal validity, and power. The TMS Quality Checklist is used to assess the methodological quality of studies that use TMS for outcome measurement. It considers 30 factors related to participant characteristics, experimental methodology, and analysis, and four factors related to paired-pulsed techniques. For each checklist, the scores were converted to percentages. Scores above 75% were deemed high quality, 50–75% moderate quality, and below 50% poor quality, as per previous publications that had utilized these checklist tools (Mani et al., 2010; Parker et al., 2016; Saywell et al., 2016).

### Data Analysis

A descriptive analysis of the results was carried out with a focus on the effect of the intervention on CME, the stimulation parameters utilized, and the methodological rigor.

## Results

### Identification and Selection of Studies

**Figure 1:**
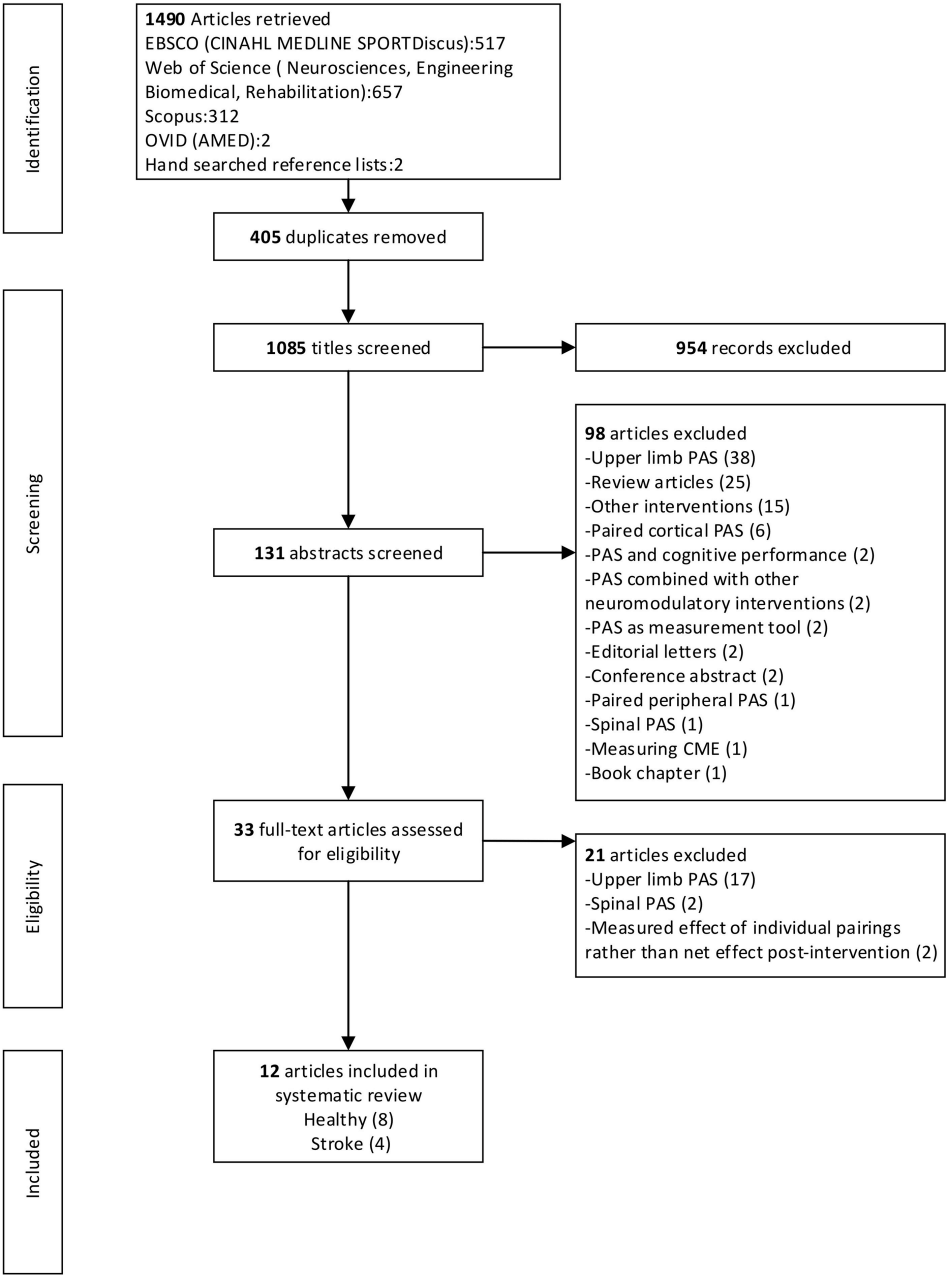
PRISMA flow chart.

After the removal of duplicates the electronic database literature search yielded 1,083 citations and a further two articles were identified from hand searching. After exclusion based on title and abstract, 33 articles were obtained for full-text review. Following the full-text review, 21 articles were excluded. A total of 12 articles, eight with healthy participants and four with participants with stroke, met the selection criteria and were included in the review. Where multiple experiments were presented in one article, all experiments were included that fulfilled the inclusion criteria. There were 24 independent experiments across the 12 articles. Figure 1 provides a flow chart that summarizes the study selection process.

### Description of Included Studies

#### Participants

A total of 150 healthy participants and 39 people with chronic stroke were included across the 12 articles. Healthy participants were younger than those with stroke (age range 19–68 years and 37–79 years, respectively). Male to female representation was similar across healthy studies (males *n* = 85, females *n* = 75), but there was a larger proportion of males in the stroke studies (males *n* = 26, females *n* = 13). Participants with stroke varied in lesion location and time since stroke (11 months to 33.1 years). Lesion side was comparable, with 21 participants with right hemiplegia and 18 with left hemiplegia.

#### Study Designs

The experiments utilized pre-post, pre-post repeated measures, and same-subject repeated measures crossover study designs (Uy et al., 2003; Stinear and Hornby, 2005; Prior and Stinear, 2006; Jayaram et al., 2007; Mrachacz-Kersting et al., 2007; Roy et al., 2007; Jayaram and Stinear, 2008, 2009; Rogers et al., 2011; Kumpulainen et al., 2012, 2015; Mrachacz-Kersting and Stevenson, 2017). The experiments explored either the efficacy of a single PAS intervention, the efficacy of different types of single-session PAS interventions with varying stimulation parameters, or made comparisons between PAS and other neuromodulatory interventions such as rTMS and tDCS. Only three articles compared PAS to a control intervention (Stinear and Hornby, 2005; Jayaram et al., 2007; Mrachacz-Kersting et al., 2007). One article evaluated the cumulative effect of multiple sessions of PAS (Uy et al., 2003). One article established the optimal ISI initially, and then went on to evaluate the repeatability of PAS using the chosen ISI (Kumpulainen et al., 2012). See Table 3 for details of each experiment.

**Table 3:** Overview of the lower limb PAS studies included in the review.

**Study design**	**Sample**	**Target muscle**	**Interventions**	**TMS measure (post intervention time points, muscle state, stimulation output)**	**Immediate effect on MEP amplitude (expressed as % change from pre-intervention)**	**Duration of effect on mean MEP amplitude (expressed as % change pre-intervention)**	**D&B QC score (%)**	**TMS QC score (%)**
**HEALTHY STUDIES**								
**Stinear and Hornby**, **2005**								
Same-subject repeated-measures crossover	*n* = 14 (24–58 yr)	TA	2 PAS interventions, 7 days apart 1. PAS+ (late swing phase treadmill walking) 2. PAS– (late swing phase treadmill walking)	Post 0, 10 min Active (late swing phase) 1.0–1.5 mV	1. ↑ 19% (and ↑23% MEP area) 2. ↓15% (and ↓12% MEP area)	10 min 1. ↑21% (and ↑19% MEP area) 2. ↓18% (and ↓15% MEP area)	61	70
Same-subject repeated-measures crossover	*n* = 4	TA	3 control conditions, 2 PAS interventions, two single sessions Control conditions given consecutively on treadmill (1. walk only, 2. PES + walk, 3. TMS + walk) followed by: 4. PAS+, or 5. PAS- (late swing phase treadmill walking). Repeated with either 4 or 5 in next session.	Post each condition and post 10 min Active (late swing phase) 1.0–1.5 mV	Controls 1, 2, 3: no difference *X:* 4.PAS+ and 5.PAS– results grouped: ↑14% from post-intervention walk measure (shows effect of PAS vs. walking control)	*X:* 4.PAS+ and 5.PAS– grouped: 10 min ↑10% from post-intervention walk measure		
Same-subject repeated-measures	*n* = 4	TA	1 PAS intervention PAS+ with voluntary DF contraction	Post 0, 10 min Active (late swing phase), 1.0–1.5 mV	Pooled time points:↑ 23%			
**Prior and Stinear**, **2006**								
Same-subject repeated-measures crossover	*n* = 10 (20–30 yr)	TA	3 PAS interventions PAS+ during treadmill walking 1. late swing phase 2. early swing phase 3. stance phase	Post 0, 10, 20, 30 min Active (late swing phase) 1.0 mV	1. Group findings not reported *X:* Re-grouped according to response “*Facilitators”* (*n* = 5) pooled time points:↑18% late swing (vs. ↓4% early swing). Max time point: ↑30% ii). “*Inhibitors”* (*n* = 5) pooled time points: ↓23% late swing (vs. ↓1% early swing. Max time point: ↓30% late swing (vs. ↑3% early swing). 2. and 3. No difference in group effect		46	68
Same-subject repeated-measures crossover	*n* = 5 (26–32 yr)	TA	2 PAS interventions PAS+ during treadmill walking 1. late swing phase 2. mid swing phase	Post 0, 10, 20, 30 min. Active (late swing phase) 1.0 mV	1. Max time point ↑25% 2. Max time point ↓13%			
**Jayaram et al.**, **2007**								
Same-subject repeated-measures crossover	*n* = 13 (29–46 yr)	TA	3 PAS interventions PAS+ during 1. sitting with high intensity TMS (120% AMth) 2. treadmill walking with high intensity TMS (120% AMth) 3. Sitting with low intensity TMS (100% AMth)	Post 0, 5, 10, 15, 20 min Active (late swing phase) 1.0 mV enditemize	No difference	*Within group:* 1. 5 min ↑12.8%, 15 min ↑16.1% 2. No difference 3. No difference *Between group:* 1 > 3 5 min (↑12.8% vs. ↓6%) 10 min (↑7.1% vs. ↓ 7.9%) 15 min (↑16.1% vs. ↓2.5%) Max time point: (↑24% vs. ↑7%) 2 > 3 5 min (↑8.5% vs. ↓6%)	50	68
**Mrachacz-Kersting et al.**, **2007**								
Same-subject pre-post crossover	*n* = 5	TA	7 PAS interventions PAS+ to inactive muscle with ISIs of 1. 20 ms 2. 30 ms 3. 40 ms 4. 45 ms 5. 50 ms 6. 55 ms 7. 60 ms	Post 30 min Resting 120% RMTh	Not tested	30 min 1. No difference 2. No difference 3. 40 ms ≈↓20% 4. 45 ms ≈↑135%, 5. 50 ms ≈↑150%, 6. 55 ms ≈↑340% 7. No difference	57	70
Same-subject repeated-measures crossover	*n* = 14	TA	2 PAS interventions, 1 control condition 1. PAS+ to inactive muscle (ISI 55 ms) 2. PAS+ with voluntary DF contraction (ISI 55 ms) 3. Voluntary DF only	Post 0 min Resting 120% RMTh	1. No difference 2. pre 0.26 ± 0.22 mV post 0.50 ± 0.39 mV (↑92%) 3. No difference	Not tested		
Same-subject repeated-measures crossover	*n* = 5	TA	2 PAS interventions PAS+ with voluntary DF contraction (ISI 55 ms) with 1. Moderate TMS intensity (120% RMTh) 2. Low TMS intensity (80-100% RMTh)	Post 0, 15, 30 min Resting 120% RMTh	1. ≈↑180% TMS 2. ≈↑175% TMS No between group differences	Not reported		
Same-subject repeated-measures	*n* = 13	TA	1 PAS intervention PAS+ to inactive muscle (ISI = SEP “N34” latency + 6 ms)	Post 0,30 min Resting 120% RMTh	↑96%	30 min ↑88%		
Pre-post	*n* = 12	TA	1 PAS intervention PAS+ to inactive muscle (ISI = SEP “N34” latency + 6 ms)	Post 0 min Resting 120% RMTh	↑67% (*n* = 5) ↑73% (*n* = 7)	Not tested		
**Roy et al.**, **2007**								
Pre-post repeated-measures overlapping but different subjects	*n* = 18 (each group *n* = 8, except control *n* = 5)	TA	6 PAS interventions, 1 control intervention PAS+ to inactive muscle ISIs adjusted based on MEP latency 1. −40 ms (TMS precedes) 2. 0 ms 3. 20 ms 4. 35 ms 5. 40 ms 6. 60 ms 7. control −170 ms (TMS precedes)	Post 0, 10, 20, 30, 60 min a) Resting 300% PTh or 150% RMTh b) Active (10% MVC) 1.0 mV	a) Resting MEPs −40ms ↑≈65% 2–7. No difference b) Active MEPs 1–7. No difference	a) Resting MEPs 1. −40 ms 10 min ↑≈60%, 20 min ↑≈55%, 60 min ↑≈65% 2. 0 ms 10 min ↑≈25%, 20 min ↑≈45%, 30 min ↑≈67%, 60 min ↑≈40% 3–7. No difference b) Active MEPs 1, 2, 5, 6. No difference *Grouped resting and active MEPS* 1,2. No difference 3. 10-60min ↑ (A&P) 4. 10-60min ↑ 5,6,7. No difference	39	69
Pre-post repeated-measures	*n* = 8	TA	1 PAS intervention PAS+ to inactive muscle with low intensity TMS (80% AMTh)	Post 0, 10, 20, 30, 60 mina) Resting 300% PTh or 150% RMTh b) Active (10% MVC) 1.0 mV	a) Resting MEPs No difference b) Active MEPs ↑≈35%	a) Resting MEPs10 min ↑≈ 30%, 20 min ↑≈55%, 30 min ↑≈70%, 60 min ↑≈85% b) Active MEPs0–60 min ↑21 ± 16%, 10 min ↑≈20%, 30 min ↑≈25%		
Pre-post repeated-measures	*n* = 8	TA	1 PAS intervention PAS+ to inactive muscle (ISI = 20 ms)	Post 15, 30 min Resting Paired pulse: conditioning 95% AMTh, test 0.3–0.6 mV	Not tested	15 min ↑41% SICI and ICF: no difference 30 min ↑37% SICI and ICF: No difference		
**Kumpulainen et al.**, **2012**								
Same-subject pre-post crossover	*n* = 8 (22–28 yr)	SOL	4 PAS interventions, 3 days apart PAS+ to inactive muscle with ISIs of SEP “P32” latency plus either 1. 6 ms 2. 12 ms 3. 18 ms 4. 24 ms	Post 5 mina) Resting b) Active (5% MVC)120% RMTh	Not tested	5 mina) Resting MEPs 1. ↓31 ± 30% (ISI 38 ± 2 ms) 2. No difference 3. ↑88 ± 105% (ISI 50 ± 2 ms) 4 No differenceb) Active MEPs: 1–4 no difference	61	60
Pre-post repeated-measures	*n* = 8 (20–24 yr)	SOL	1 PAS intervention PAS+ to inactive muscle (ISI = SEP “P32” latency + 18 ms)	Post 0, 30 min Restinga) 90, 100, 110, 120, 130, 140% RMThb) mean of (a) above	a) stimulus response curve no change ↑43 ± 44%	30 min a) stimulus response curve slope↑73%b) ↑53 ± 41%		
Same-subject pre-post	*n* = 8 (23–27 yr)	SOL	1 PAS intervention, repeated twice ≥3 days apart PAS+ to inactive muscle (ISI = SEP “P32” latency + 18 ms)	Post 5 mina) Restingb) Active (5% MVC)120% RMTh	Not tested	5 min. a) Resting MEPs:↑46 ± 52%, ↑36 ± 32% (session 1, 2)b) Active MEPs: No difference		
**Kumpulainen et al.**, **2015**								
Pre-post repeated-measures	*n* = 30 (21–29 yr)	SOL	2. PAS interventions, 2 groups 3. PAS+ with voluntary PF contraction (ISI = 50 ms) (*n* = 15) 2. PAS- with voluntary PF contraction (ISI = 20 ms) (*n* = 15)	Post 0, 15 min a) Resting 120% RMTh b) Active (20% MV)120% AMTh c) Active (50% MVC)120% AMTh	1. a) No difference (*X:* Grouped 11 responders ↑≈87%) b) No difference c) ↓9 ± 12% 2. a) ↓27 ± 32% b) ↓15 ± 25% c) No difference	15 min. 1. a) ↑73 ± 123%b) No differencec) ↓8 ± 14% 2. a) No difference(*X:* Grouped 12 responder's ↓≈43%)b) ↓9 ± 18%c) No difference	61	65
**Mrachacz-Kersting and Stevenson**, **2017**								
Same-subject repeated-measures crossover	*n* = 11 (22–32 yr)	TA	2 PAS interventions, 7 days apart PAS+ to inactive muscle (ISI = SEP “N34” latency +6 ms) 1. Biphasic TMS pulse 2. Monophasic TMS pulse	Post 0, 30 min Resting 120% RMTh	1. pre 0.32 ± 0.23 mV post 0.43 ± 0.24 mV (↑74%) 2. pre 0.28 ± 0.14 mV post 0.44 ± 0.22 mV (↑83%) No between group differences	1.30 min 0.60 ± 0.33 mV(↑117%) 2. 30 min 0.49 ± 0.18 mV(↑105%) No between group differences	57	74
Same-subject pre-post crossover	*n* = 10 (22–28 yr)	TA	2 PAS interventions, ≥7 days apart PAS+ to inactive muscle (ISI = ISI = SEP “N34” latency +6 ms 1. High intensity TMS (120% RMTh) 2. Low intensity TMS (95% AMTh)	Post 0,30 minResting120% RMTh	1. pre 0.37 ± 0.26 mV post 0.46 ± 0.22 mV (↑53%) 2. pre 0.39 ± 0.29 mV post 0.50 ± 0.39 mV (↑55%) No between group differences	1.30 min 0.62 ± 0.37 mV(↑95%) 2. 30 min 60 ± 0.47 mV(↑80%) No between group differences		
**STROKE STUDIES**								
**Uy et al.**, **2003**								
Pre-post uncontrolled	*n* = 9 (43–78 yr)	TA and PL	1. PAS intervention PAS+ to inactive muscle (ISI = 35 ms) 20 sessions over 4 weeks	Post 4 weeksa) Resting 115% RMTh b) Active (5-10% MVC) 115% AMTh	Not tested	4 weeks No difference*X:* a) *n* = 5 ↑TA, *n* = 3 ↑PLb) *n* = 3 ↑TA, *n* = 5 ↑PL	38	38
**Jayaram and Stinear**, **2008**								
Pre-post repeated-measures	People with chronic stroke (*n* = 10) (44–64 yr) Age-matched healthy (*n* = 10)	TA	1 PAS intervention, 2 groups PAS– to inactive muscle (ISI = MEP latency minus 8 ms) Stroke: Applied to contralesional hemisphere and non-paretic TA Healthy: Applied to dominant hemisphere and contralateral TA	Post 0, 5, 10, 15 min Active (late swing phase) mV Post 0, 5, 10, 15 min Active (late swing phase) 1.0 mV	Paretic TA: ↑34% Non-paretic TA: No difference Non-stimulated TA: ↑18% Stimulated TA: No difference	Paretic TA: 5 min ↑30%, 10 min ↑20%, 15 min ↑38% Pooled time points: ↑30% Non-paretic TA: No difference Pooled time points: ↓9% Non-stimulated TA: 5 min ↑32%, 10 min ↑23%, 15 min ↑34% Pooled time points:↑26% Stimulated TA: 10 min ↓15%, 15 min ↓16% Pooled time points: ↓13%	64	75
**Jayaram and Stinear**, **2009**								
Same-subject repeated-measures crossover	People with chronic stroke (*n* = 9) (45–66 yr)	TA	3 interventions, 2 days apart 1. PAS– (late swing phase treadmill walking, ISI = MEP latency minus 8 ms). Contralesional hemisphere and non-paretic TA 2. rTMS– contralesional 3. tDCS+ ipsilesional	Post 0, 10, 20 min TA: Active (late swing phase) 120% AMTh MH, VL, and MG: Peak activation during gait cycle 120% AMTh	Non-stimulated: pooled time points TA ↑20%, MH VL MG no difference Stimulated limb: pooled time points MH ↓24%, TA VL MG No difference Non-stimulated: max time point TA ↑22%, MH VL MG No difference Stimulated limb: max time point MH ↓37%, TA VL MG No difference		56	73
**Rogers et al.**, **2011**								
Pre-post repeated-measures	People with chronic stroke (*n* = 11) (37–79 yr)	VM	1 PAS intervention, 2 groups Stroke: PAS– to inactive muscle, contralesional hemisphere and non-paretic VM	Post 0, 10, 20, 30 min Active (mid VM burst on cycle ergometer), MEP size 2–3 times background EMG	Paretic VM: No difference Non-paretic VM: ↓	Paretic VM: No difference Non-Paretic VM: 10 min ↓, 20 min ↓, 30 min ↓ Pooled time points: ↓21%.	58	65
	Healthy (*n* = 11) (27–69 yr)		Healthy: PAS– to inactive muscle, left hemisphere and right VM	Post 0, 10, 20, 30 min Active (mid VM burst on cycle ergometer), MEP size 2–3 times background EMG	Non-stimulated: No difference Stimulated VM: ↓	Non-stimulated VM: 10, 20–30 min No difference Stimulated VM: 10 min ↓ 20–30 min No difference Pooled time points ↓15%		

*vs, compared with another group for statistical significance; MEP value is expressed as relative % change from pre-intervention where 0% = no change; ≈, approximated from graphs presented in article; ↓, significantly decreased MEP (% or mV) alpha 0.05;↑, significantly increased MEP (% or mV) alpha 0.05; Pooled time points, all post-intervention time points pooled for analysis; Max time point, time point with maximum facilitation used for analysis; AMth, active motor threshold; CPN, common peroneal nerve; D&B QC, Modified Downs and Black Quality Checklist; DF, dorsiflexion; FN, femoral nerve; ICF, intracortical facilitation; ISI, inter stimulus interval (unless stated otherwise, peripheral electrical stimulation is delivered first); MEP, motor evoked potential; MEPmax, maximum MEP amplitude; MG, medial gastrocnemius muscle; MH, medial hamstrings muscle; MVC, maximum voluntary contraction; PAS+, facilitatory PAS; PAS–, inhibitory PAS; PES, peripheral electrical stimulation; PL, peroneus longus muscle; PTh, perceptual threshold; RMTh, resting motor threshold; Rpm, revolutions per minute; rTMS, repetitive transcranial magnetic stimulation; SEP, somatosensory evoked potential; SICI, short interval intracortical inhibition; SOL, soleus muscle; STh, sensory threshold; TA, tibialis anterior muscle; TDCS, Transcranial direct current stimulation TMS, transcranial magnetic stimulation; TMS QC, TMS Quality Checklist; TN, tibial nerve; VL, vastus lateralis muscle; VM, vastus medialis muscle; X, unplanned analysis or unconventional analysis*.

#### PAS Interventions

Among the 12 articles there were 20 experiments in healthy people (*n* = 136) and one experiment in people with stroke (*n* = 9) that investigated facilitatory PAS interventions; these targeted the corticospinal pathway of either the tibialis anterior muscle (TA) (Uy et al., 2003; Stinear and Hornby, 2005; Prior and Stinear, 2006; Jayaram et al., 2007; Mrachacz-Kersting et al., 2007; Roy et al., 2007; Mrachacz-Kersting and Stevenson, 2017), the peroneous longus muscle (PL) (Uy et al., 2003), or the soleus muscle (SOL) (Kumpulainen et al., 2012, 2015). The target muscle was either inactive (Uy et al., 2003; Jayaram et al., 2007; Mrachacz-Kersting et al., 2007; Roy et al., 2007; Kumpulainen et al., 2012, 2015; Mrachacz-Kersting and Stevenson, 2017), engaged in a voluntary contraction (Prior and Stinear, 2006; Mrachacz-Kersting et al., 2007; Kumpulainen et al., 2015), or activated during treadmill walking (Stinear and Hornby, 2005; Prior and Stinear, 2006). Inhibitory PAS interventions were investigated in three experiments in healthy people (*n* = 50); two targeting the TA muscle during treadmill walking (Stinear and Hornby, 2005) and one targeting the SOL muscle during voluntary contraction (Kumpulainen et al., 2015). Three experiments in people with stroke (*n* = 30) applied inhibitory PAS to the contralesional hemisphere to decrease asymmetrical inter-hemispheric inhibition; these targeted the non-paretic TA (Jayaram and Stinear, 2008, 2009) or vastus medialis muscle (VM) (Rogers et al., 2011). Two of these experiments also included healthy controls (*n* = 21) (Jayaram and Stinear, 2008; Rogers et al., 2011).

#### Measurement Outcomes

All experiments measured CME with single pulse TMS (Uy et al., 2003; Stinear and Hornby, 2005; Prior and Stinear, 2006; Jayaram et al., 2007; Mrachacz-Kersting et al., 2007; Roy et al., 2007; Jayaram and Stinear, 2008, 2009; Rogers et al., 2011; Kumpulainen et al., 2012, 2015; Mrachacz-Kersting and Stevenson, 2017). Changes in TMS-induced MEP amplitude (or area) were expressed as either a relative percentage change (pre-intervention value normalized to 100%) and or an absolute mean change (mV). Some experiments also measured MEP amplitude stimulus response curves (Kumpulainen et al., 2015) or intracortical facilitation and inhibition (Roy et al., 2007). TMS measurements were recorded from a target muscle in its resting state (Uy et al., 2003; Mrachacz-Kersting et al., 2007; Roy et al., 2007; Kumpulainen et al., 2012, 2015; Mrachacz-Kersting and Stevenson, 2017), during an active contraction (Uy et al., 2003; Roy et al., 2007; Kumpulainen et al., 2012, 2015), during treadmill walking (Stinear and Hornby, 2005; Prior and Stinear, 2006; Jayaram et al., 2007; Jayaram and Stinear, 2008, 2009), or whilst pedaling on a static cycle (Rogers et al., 2011).

### Methodological Quality Assessment

Refer to Table 3 for quality scores, and Supplementary Material for breakdown of scores. Assessors examining methodological quality demonstrated excellent pre consensus interrater reliability for both quality tools (Downs and Black κ = 0.896, TMS Quality checklist κ = 0.931). The Downs and Black quality checklist revealed that articles were of low to moderate quality (mean overall score 54%, SD 8%, range 38–64%). In general, authors failed to control for confounding variables, adverse events, external validity, blinding, selection bias, and power. The TMS Quality Checklist tool revealed an overall mean quality score of 66% (SD 9%; range 38–75%), with all but one article (Uy et al., 2003) being deemed moderate quality. For the majority of studies, the TMS method and analysis was well-described, but information about certain factors that can influence MEP measurement was lacking (medications, medical comorbidities, participation in repetitive motor activity, target muscle activity prior to TMS stimulation, and activity of surrounding muscles).

### Intervention Efficacy

Intervention efficacy was determined based on statistically significant changes in group mean MEP amplitude (or area) of the target muscle. Approximate changes (≈) have been interpreted from graphs where mean and variance estimates were not provided, and as such should be interpreted with caution. A summary of the main results can be found in Table 3.

#### Immediate Changes

Of the 12 experiments in healthy people that analyzed the immediate effects of facilitatory PAS, 10 reported statistically-significant increases in relative mean MEP amplitude immediately post-intervention (range 19 to ≈180%; Stinear and Hornby, 2005; Mrachacz-Kersting et al., 2007; Roy et al., 2007; Kumpulainen et al., 2012; Mrachacz-Kersting and Stevenson, 2017). Three experiments reported no statistically-significant differences (Jayaram et al., 2007; Mrachacz-Kersting et al., 2007; Kumpulainen et al., 2015). An additional four experiments in healthy people recorded MEPs immediately following facilitatory PAS, but grouped the data with other post-intervention time-points (Stinear and Hornby, 2005; Prior and Stinear, 2006; see duration of effect section below) or with inhibitory PAS data (Stinear and Hornby, 2005), or failed to report the primary outcome of MEP amplitude (Prior and Stinear, 2006). Most facilitatory PAS experiments (*n* = 16 of total 20) targeted the TA. The immediate relative effect was largest when PAS was delivered during a TA voluntary contraction using an ISI of 55 ms or an optimized ISI based on individualized common peroneal nerve SEP latency (N34 peak), and when recording MEPs from the TA at rest (Mrachacz-Kersting et al., 2007; Mrachacz-Kersting and Stevenson, 2017). For the SOL muscle, immediate relative increases in CME occurred when PAS was delivered to an inactive muscle and MEPs were recorded from the SOL at rest (Kumpulainen et al., 2012). In contrast, facilitatory PAS delivered to the SOL muscle during a small voluntary contraction did not increase resting or active MEPs; however when the authors undertook an additional unplanned analysis excluding non-responders (*n* = 4 out of 15), there was a significant increase in resting MEPs (Kumpulainen et al., 2015).

Four experiments analyzed the immediate effects of inhibitory PAS (Stinear and Hornby, 2005; Jayaram and Stinear, 2008; Kumpulainen et al., 2015) One additional healthy experiment studied inhibitory PAS, but as referred to above, grouped the effects of inhibitory and excitatory PAS together (Stinear and Hornby, 2005). Across healthy participants, there were immediate relative decreases in CME when inhibitory PAS was applied to: the TA during treadmill walking (Stinear and Hornby, 2005; Jayaram and Stinear, 2008), the SOL muscle during a small voluntary contraction (Kumpulainen et al., 2015), and the inactive vastus medialis muscle (VM) (Rogers et al., 2011). The three experiments involving participants with stroke applied inhibitory PAS to the unaffected hemisphere and inactive non-paretic target muscle, and showed increased relative TA MEP amplitudes of the paretic TA (recorded during treadmill walking; Jayaram and Stinear, 2008, 2009), but no effect in the affected VM (recorded during pedaling on a static cycle; Rogers et al., 2011).

#### Duration of Effect

Thirteen experiments in healthy people reported the duration of effect following facilitatory PAS. Increases in CME were observed from 5 to 60 min post intervention and ranged from relative increases of 13 to ≈340% (Stinear and Hornby, 2005; Jayaram et al., 2007; Mrachacz-Kersting et al., 2007; Roy et al., 2007; Kumpulainen et al., 2012, 2015; Mrachacz-Kersting and Stevenson, 2017). An additional four experiments collected MEPs at various post-intervention time-points but either: grouped all post-intervention time- points (Stinear and Hornby, 2005), combined results with inhibitory PAS experiments (Stinear and Hornby, 2005), didn't report the primary outcome (Prior and Stinear, 2006), or only analyzed the time-point of maximum facilitation (Prior and Stinear, 2006).

The largest MEP increases were observed at 30 min post-intervention when facilitatory PAS was delivered to the inactive TA, using an ISI of 55 ms or individualized to SEP latency (N34), and MEPs were recorded from the TA at rest (Mrachacz-Kersting et al., 2007; Mrachacz-Kersting and Stevenson, 2017). When PAS was delivered with low TMS (80% AMTh), increases in excitability were observed up to an hour post-intervention in both resting and active MEPs (≈85 and ≈25%, respectively; Roy et al., 2007). Of the four facilitatory experiments that targeted the SOL muscle, increases in MEP amplitude were shown at 5, 15, and 30 min post-intervention (Kumpulainen et al., 2012, 2015). MEP increases were largest 5 min post-intervention, using a PAS protocol applied to the inactive muscle with an ISI equal to “SEP latency (P32) + 18 ms” (mean 50 ± 2 ms) (mean increase 88%; Kumpulainen et al., 2012). Based on these results, a later study set the ISI at 50 ms and delivered PAS during a small plantar flexor contraction [5% maximum voluntary contraction (MVC)]; a facilitatory effect was measured 15 min post-intervention (mean increase 73%; Kumpulainen et al., 2015).

With regards to inhibitory PAS, four experiments assessed the duration of effect at a range of time-points post-intervention, and showed an inhibitory effect at 10–15 min post-intervention in healthy people (Stinear and Hornby, 2005; Jayaram and Stinear, 2008; Rogers et al., 2011; Kumpulainen et al., 2015) and 10–30 min post-intervention in people with stroke (Jayaram and Stinear, 2008; Rogers et al., 2011). One additional experiment, with both healthy and stroke participants, investigated the effects of inhibitory PAS at 10 and 20 min post-intervention, but performed statistical analysis on only a combination of both time-points and the point of maximum modulation (Jayaram and Stinear, 2009). Another study combined results following inhibitory PAS with excitatory PAS (Stinear and Hornby, 2005). Across the healthy experiments, small decreases in MEP amplitude were seen at 10 min following a PAS intervention delivered during treadmill walking (Stinear and Hornby, 2005), at 15 min following a PAS intervention delivered during a small plantar flexor contraction (this affected active but not resting MEPs; Kumpulainen et al., 2015), and 10–15 min following a PAS intervention delivered to the inactive TA and VM (Jayaram and Stinear, 2008; Rogers et al., 2011). In participants with stroke, inhibitory PAS applied to the unaffected hemisphere resulted in small increases in CME in the paretic TA 5–20 min post-intervention (recorded during treadmill walking; Jayaram and Stinear, 2008, 2009) but no excitation in the paretic VM 10–30 min post-intervention (recorded during pedaling; Rogers et al., 2011).

#### Effect of Multiple Sessions

When two facilitatory PAS interventions were delivered to healthy people at least 3 days apart, there were comparable increases in CME following each intervention, although cumulative effects were not explicitly explored (Kumpulainen et al., 2012). The single study which has specifically explored the cumulative effects of PAS, delivered 20 interventions over 4 weeks to people with chronic stroke (*n* = 9) and reported no statistically significant group changes in either active or resting MEPs (Uy et al., 2003).

#### Control Experiments

Four healthy experiments compared PAS to a control intervention. Controlled interventions included PES only whilst sitting, TMS only whilst sitting (Jayaram et al., 2007), treadmill walking only, TMS while treadmill walking, PES while treadmill walking (Stinear and Hornby, 2005), and dorsiflexion only in sitting (Mrachacz-Kersting et al., 2007). None of these significantly modulated CME (*p* > 0.05).

### Stimulation Parameters

PAS stimulation parameters varied across the experiments, including stimulation location, number of stimulation pairings, ISI, and the stimulation intensity, frequency, and waveform. A summary of the stimulation parameters employed in the experiments are presented in Table 4.

**Table 4:** Overview of the stimulation parameters employed across the included studies.

**Target muscle**	**Stimulus location**	**Muscle state during PAS intervention**	**ISI (ms)**	**TMS coil type, orientation and direction of induced current**	**TMS intensity**	**PES intensity**	**Number of stimulation pairings**	**Stimulation period (mins)**	**Frequency**	**Pulse width (ms)**
**HEALTHY STUDIES**										
**Stinear and Hornby**, **2005**										
TA	CPN	PAS+ - Active: treadmill walking - Active: with voluntary DF contraction	Individualized MEP latency +5 ms	- Double-cone coil - Mid-sagittal plane ≈ 1 cm anterior to the vertex - Direction of current: NR	120% AMTh	120% MTh	120	10	NR	NR
TA	CPN	PAS– - Active: treadmill walking	Individualized MEP latency −10 ms		120% AMTh	120% MTh	120	10	NR	NR
**Prior and Stinear**, **2006**										
TA	CPN	PAS+ Active: treadmill walking	Individualized MEP latency +5 ms	- Double-cone coil - Mid-sagittal plane, coil intersection ≈ 2 cm posterior to vertex - Posterior-anterior current	120% AMTh	120% MTh	120	10	0.2	1
**Jayaram et al.**, **2007**										
TA	CPN	PAS+ Inactive muscle	MEP latency +5 ms	- Double-cone coil - Mid-sagittal plane coil, intersection ≈ 2 cm posterior to vertex - Posterior-anterior current	120% AMTh	120% MTh	120	4	0.5	1
TA	CPN	PAS+ Inactive muscle	MEP latency +5 ms		100% AMTh	120% MTh	120	4	0.5	1
TA	CPN	PAS+ Active: treadmill walking	MEP latency +5 ms		120% AMTh	120% MTh	120	4	0.5	1
**Mrachacz-Kersting et al.**, **2007**										
TA	CPN	PAS+ Inactive muscle	20, 30, 40, 45, 50, 55, 60 ms	- Double-cone coil - ≈2–3 cm anterior to the vertex - Posterior-anterior current	120% RMTh	100% MTh	360	30	0.2	1
TA	CPN	PAS+- Inactive muscle - Active: 5–10% MVC	55 ms		120% RMTh	100% MTh	360	30	0.2	1
TA	CPN	PAS+ Active: 5–10% MVC	55 ms		DF matched reduced to 80-100% RMTh	100% MTh	360	30	0.2	1
TA	CPN	PAS+ Inactive muscle	Individualized SEP latency (N34) +6 ms		120% RMTh	100% MTh	360	30	0.2	1
TA	CPN	PAS+ Inactive muscle	Individualized SEP latency (N34) +6 ms		120% RMTh	100% MTh	360	30	0.2	1
**Roy et al.**, **2007**										
TA	CPN	PAS+ Inactive muscle	MEP latency −70, −30, −10, +5, +30 ms (afferent volley arrived 15–90 ms post TMS)	Double-cone coil ≈1 cm lateral and 1 cm posterior to the vertex - Posterior-anterior current	MEPs 0.3–0.6 mV	300% PTh or 150% MTh	90	15	0.1	1
TA	CPN	PAS+ Inactive muscle	15–35 ms		80% AMTh	300% STh	60	5	0.2 (X 3 stimuli at 100 Hz 10 ms train)	1
TA	CPN	PAS+ Inactive muscle	20 ms		MEPs 0.3– 0.6 mV	300% PTh or 150% MTh	90	15	0.1	1
**Kumpulainen et al.**, **2012**										
SOL	TN	PAS+ Inactive muscle	Individualized SEP latency (P32) + 6, 12, 18, 24 ms	- Double batwing coil - Optimally positioned, where SOL MEPs were greater/more consistent than MEPs of adjacent coordinates for a given stimulus intensity	120% RMTh	150% MTh	200	<20	0.2	NR
	TN	PAS+ Inactive muscle	Individualized SEP latency (P32) + 18 ms	- Direction of current: NR	120% RMTh	150% MTh	200	<20	0.2	NR
	TN	PAS+ Inactive muscle	Individualized SEP latency (P32) + 18 ms		120% RMTh	150% MTh	200	<20	0.2	NR
**Kumpulainen et al.**, **2015**										
SOL	TN	PAS+ Active: 5% MVC	50 ms	- Double batwing coil - ≈1 cm lateral and 1 cm posterior to the vertex	120% RMTh	150% MTh	200	17	0.2	NR
SOL	TN	PAS– Active: 5% MVC	20 ms	- Direction of current: NR	120% RMTh	150% MTh	200	17	0.2	NR
**Mrachacz-Kersting and Stevenson**, **2017**										
TA	CPN	PAS+ Inactive muscle	Individualized SEP latency (N34) +6 ms	- Double-cone coil - ≈1 cm lateral and 1 cm posterior to the vertex - Posterior-anterior current	120% RMTh	100% MTh	360	30	0.2 biphasic pulse	1
TA	CPN	PAS+ inactive muscle	Individualized SEP latency (N34) +6 ms		120% RMTh	100% MTh	360	30	0.2 Monophasic pulse	1
TA	CPN	PAS+ Inactive muscle	Individualized SEP latency (N34) +6 ms		−95% AMTh - 120% RMTh	100% MTh	360	30	0.2 biphasic pulse	1
**STROKE STUDIES**										
**Uy et al.**, **2003**										
TA/PN	CPN	PAS+ Inactive muscle	35 ms	- Angled figure of eight coil - Region of the vertex - Direction of current: NR	100% MTh	100% MTh	180	30 4 weeks	0.1 (PES: 500 ms train of 10 Hz)	1
**Jayaram and Stinear**, **2008**										
TA	CPN	PAS– Inactive muscle	Individualized MEP latency −8 ms	- Double-cone coil - Mid-sagittal plane coil, intersection ≈ 2 cm posterior to vertex - Posterior-anterior current	120% AMTh	120% MTh	120	4	NR	1
**Jayaram and Stinear**, **2009**										
TA	CPN	PAS– Active: treadmill walking	Individualized MEP latency −8 ms	- Double-cone coil - Mid-sagittal plane coil, intersection ≈ 2 cm posterior to vertex - Direction of current: NR	120% AMTh	120% MTh	120	4	0.2	1
**Rogers et al.**, **2011**										
VM	FN	PAS– Inactive muscle	Individualized MEP latency −8 ms	- Double-cone coil - Mid-sagittal plane coil, intersection ≈ 2 cm posterior to vertex - Posterior-anterior current	Active (mid VM burst on cycle ergometer), MEP size 2-3x background EMG	120% MTh	120	≈11	0.5	NR

*AMth, active motor threshold; ≈, approximately; ≈, approximate time in minutes; CPN, common peroneal nerve; DF, dorsiflexion; FN, femoral nerve; Hz, hertz; ISI, inter stimulus interval (unless stated otherwise, peripheral electrical stimulation is delivered first); MG, medial gastrocnemius muscle; MEP, motor evoked potential; MEPmax, maximum MEP amplitude; ms, milliseconds; MTh, motor threshold; MVC, maximum voluntary contraction; mV, millivolts; mins, minutes; NR, not reported; PAS+, facilitatory PAS; PAS–, inhibitory PAS; RMTh, resting motor threshold; PES, peripheral electrical stimulation; PL, peroneus longus muscle; PTh, perceptual threshold; SEP, somatosensory evoked potential; secs, seconds; STh, sensory threshold; SOL, soleus muscle; TA, tibialis anterior muscle; TMS, transcranial magnetic stimulation; TN, tibial nerve; VM, vastus medialis muscle*.

#### Stimulation Location and Application

The peripheral electrical stimulation component of the PAS targeted the TA muscle via the common peroneal nerve (CPN) in 19 experiments (Uy et al., 2003; Stinear and Hornby, 2005; Prior and Stinear, 2006; Jayaram et al., 2007; Mrachacz-Kersting et al., 2007; Roy et al., 2007; Jayaram and Stinear, 2008, 2009; Mrachacz-Kersting and Stevenson, 2017), the SOL muscle via the tibial nerve (TN) in four experiments (Kumpulainen et al., 2012, 2015), and the vastus medialis (VM) muscle via the femoral nerve (FN) in one experiment (Rogers et al., 2011). The TMS component of the PAS was delivered over the M1 at the optimal stimulation site for the target muscle, with the exception of 12 experiments that chose a site where MEP outcomes could be elicited from both the target muscle and its antagonist (Mrachacz-Kersting et al., 2007; Mrachacz-Kersting and Stevenson, 2017) and the contralateral side (Stinear and Hornby, 2005; Prior and Stinear, 2006; Jayaram and Stinear, 2009). The type of TMS coil employed and its orientation was reported for all experiments. Eighteen applied a double-cone coil (Stinear and Hornby, 2005; Prior and Stinear, 2006; Jayaram et al., 2007; Mrachacz-Kersting et al., 2007; Roy et al., 2007; Jayaram and Stinear, 2008, 2009; Rogers et al., 2011; Mrachacz-Kersting and Stevenson, 2017), four a batwing coil (Kumpulainen et al., 2012, 2015) and one experiment described an angled figure of eight coil (Uy et al., 2003). Seventeen experiments reported the direction of the TMS current flow across the cortex, all of which reported a posterior-anterior current flow (Prior and Stinear, 2006; Jayaram et al., 2007; Mrachacz-Kersting et al., 2007; Jayaram and Stinear, 2008; Rogers et al., 2011; Mrachacz-Kersting and Stevenson, 2017).

#### Dose: Number of Stimulation Pairings and Intervention Duration

Across the experiments, PAS interventions included 60–360 stimulation pairings lasting 4–30 min. Results varied across studies. To compare whether the number of stimulation pairings and duration of stimulation effected CME, one experiment recorded MEPs midway through, immediately following, and 30 min following a facilitatory PAS intervention (Mrachacz-Kersting et al., 2007). After 180 pairings there was no statistically-significant increase in resting MEP amplitude, but after 360 pairings a significant effect was observed, and this was maintained at 30 min post-intervention. In contrast, another experiment delivered a relatively short 5 min intervention, with 60 pairings (3 afferent stimuli to 1 TMS stimulation), and showed increases in resting MEP amplitudes of ≈30–85% from 10 to 60 minutes post-intervention (Roy et al., 2007). Across all experiments, those that delivered more stimulation pairings for longer periods to an inactive target muscle tended to yield greater effects (Mrachacz-Kersting et al., 2007; Roy et al., 2007; Kumpulainen et al., 2012, 2015) than those which delivered fewer stimulation pairings for shorter periods to a muscle that was either inactive or engaged in treadmill walking (Stinear and Hornby, 2005; Prior and Stinear, 2006; Jayaram et al., 2007; Jayaram and Stinear, 2009).

#### Interstimulus Interval (ISI)

ISIs were estimated based on either MEP latencies (Stinear and Hornby, 2005; Prior and Stinear, 2006; Jayaram et al., 2007; Jayaram and Stinear, 2008, 2009; Rogers et al., 2011), SEP latencies (either the N34 or P32 peaks; Mrachacz-Kersting et al., 2007; Kumpulainen et al., 2012; Mrachacz-Kersting and Stevenson, 2017), or on findings from previous lower limb PAS experiments (Mrachacz-Kersting et al., 2007; Roy et al., 2007; Kumpulainen et al., 2012, 2015). Across 18 experiments, facilitatory effects were seen when ISIs ranged from 33.5 to 56 ms for the TA (Stinear and Hornby, 2005; Prior and Stinear, 2006; Jayaram et al., 2007; Mrachacz-Kersting et al., 2007; Mrachacz-Kersting and Stevenson, 2017) and 48–52 ms for the SOL (Kumpulainen et al., 2012, 2015). In five experiments, inhibitory effects were seen when ISIs were in the range of 18–24 ms for the TA (Stinear and Hornby, 2005; Jayaram and Stinear, 2008, 2009), 16–18 ms for the VM (Rogers et al., 2011), and 20 ms for SOL (Kumpulainen et al., 2015). The largest increases in CME were seen in the TA muscle with ISIs of 40–55 ms (Mrachacz-Kersting and Stevenson, 2017). It should be noted that when ISIs were individualized to SEP latency, all participants demonstrated increased CME (Mrachacz-Kersting et al., 2007; Mrachacz-Kersting and Stevenson, 2017). In contrast, one paper showed facilitatory effects with a wide range of ISIs (−40, 0, 20, and 35 ms) where the peripheral electrical stimulus (PES) was timed to arrive up to 90 ms after the TMS (Roy et al., 2007).

#### Stimulation Intensity

Across the experiments, a range of TMS intensities for the PAS interventions were described. The most common TMS intensity, used in 11 experiments, was 120% of resting motor threshold (RMTh) with an inactive (Mrachacz-Kersting et al., 2007; Kumpulainen et al., 2012; Mrachacz-Kersting and Stevenson, 2017), or slightly contracted (Kumpulainen et al., 2015) target muscle. Six experiments used a TMS intensity of 120% of active motor threshold (AMTh) during treadmill walking (Stinear and Hornby, 2005; Prior and Stinear, 2006; Jayaram and Stinear, 2009). Other intensities can be seen in Table 4. Several experiments indicated that PAS yielded similar increases in CME when higher and lower TMS intensities were utilized (80–100% RMTh vs. 120% RMTh; 95% AMTh vs. 120% RMTh; 80% AMTh) and MEPS were recorded from a resting muscle (Mrachacz-Kersting et al., 2007; Mrachacz-Kersting and Stevenson, 2017) and a slighted contraction muscle (Roy et al., 2007). However, when a lower TMS intensity (100% AMTh obtained during treadmill) applied in sitting was compared with higher intensities applied in sitting or during walking (120% AMTh obtained during treadmill walking), and MEPS were recorded during treadmill walking, results were significantly better with both high intensity TMS conditions (Jayaram et al., 2007).

The intensities of PES varied across the experiments and were calculated as proportions of either motor threshold (MTh), sensory threshold (STh), or perceptual threshold (PTh). When PAS was applied to the inactive muscle, intensities of peripheral stimulation included 100% MTh (Uy et al., 2003; Mrachacz-Kersting et al., 2007; Mrachacz-Kersting and Stevenson, 2017), 120% MTh (Jayaram et al., 2007; Jayaram and Stinear, 2008; Rogers et al., 2011), 150% MTh (Kumpulainen et al., 2012), and either 300% STh/PTh or 150% MTh (Roy et al., 2007). When PAS was applied to the active muscle, PES intensities of 100% MTh (Mrachacz-Kersting et al., 2007) and 150% MTh (Kumpulainen et al., 2015) were used during a voluntary contraction, and 120% MTh was used during treadmill walking (Stinear and Hornby, 2005; Prior and Stinear, 2006; Jayaram et al., 2007; Jayaram and Stinear, 2009). No studies provided a rationale for the PES intensity used, nor compared the effect of different intensities. There were no apparent differences between facilitatory and inhibitory PAS interventions, nor healthy or stroke experiments in relation to the PES intensity utilized.

#### Stimulation Frequency

The frequency at which each pair of PES and TMS were paired together was reported across the majority of experiments and ranged from 0.1 to 0.5 HZ (Uy et al., 2003; Prior and Stinear, 2006; Jayaram et al., 2007; Mrachacz-Kersting et al., 2007; Roy et al., 2007; Jayaram and Stinear, 2008, 2009; Kumpulainen et al., 2012, 2015; Mrachacz-Kersting and Stevenson, 2017). Two studies also reported the frequency of PES because they matched multiple afferent stimuli with each TMS pulse (Uy et al., 2003; Roy et al., 2007). Of these studies, one showed no significant change in resting or active MEPs following a 4-week intervention (Uy et al., 2003), whilst the other demonstrated an increase in both resting and active MEPs, lasting 60, and 30 min, respectively (Roy et al., 2007). The literature did not allow a direct comparison of the effect of multiple vs. single afferent stimuli due to the variation in other intervention parameters across studies.

#### PES Pulse Width

Twenty one experiments specified that the peripheral stimulation was delivered with a pulse width of 1 ms; however justification for this was not provided. No experiments compared the effects of different pulse widths.

#### Pulse Waveform

Only six experiments with healthy people and one experiment with people with stroke documented the waveform of the TMS pulse; six utilized a monophasic pulse and two a biphasic pulse (Jayaram and Stinear, 2009; Kumpulainen et al., 2012, 2015; Mrachacz-Kersting and Stevenson, 2017). When PAS interventions with either mono- or bi-phasic waveforms were compared, both were equally effective (Mrachacz-Kersting and Stevenson, 2017). With regards to the PES, waveform selection was not reported across any of the experiments.

## Discussion

This systematic review examined the efficacy of PAS on lower limb CME in healthy and stroke populations, whilst explicitly considering the methodological quality of the research and the influence of stimulation parameters on the reported outcomes. Bearing in mind that the body of literature lacks methodological rigor and therefore may be subject to bias, the key finding supports the efficacy of a single session of lower limb PAS to modulate CME in healthy and stroke populations. An important limitation is the lack of studies that have investigated the effect of multiple sessions of PAS over time, as would commonly be done in a clinical setting. Whilst stimulation parameters appear important, the ability to draw robust conclusions about the selection of optimal parameters is hindered by: (1) limited systematic evaluation of stimulation parameter settings within and across experiments; (2) variability in the muscle state during PAS interventions; (3) inter-individual variability in the magnitude of response to the PAS interventions; and (4) variability in the muscle contraction state during CME measurement and the influence this has on the ability to elucidate an effect. We discuss the key findings below, whilst highlighting limitations in the evidence-base, and providing recommendations for future lower limb PAS research in healthy and stroke populations.

### Methodological Quality

Lower limb PAS interventions are part of an emergent field of neuromodulation research, which predominately consists of early exploratory work. The majority of studies included in this review were of moderate-to-poor quality (Downs and Black mean 54%, SD 8%, range 38-64%). Given the lack of methodological rigor in the research, care should be exercised when interpreting the findings in relation to both efficacy and optimal parameters. Study sample sizes were generally low, with only one study reporting a power calculation (Stinear and Hornby, 2005). Aspects of external validity were poorly addressed with samples often not representative of the population. All studies scored poorly on aspects of internal validity; this frequently related to a failure to consider and report relevant covariates, and the absence of both randomization and blinding of participants and assessors.

### Intervention Efficacy of Lower Limb PAS in Healthy People

The majority of studies in this review reported that a single session of facilitatory or inhibitory lower limb PAS resulted in an immediate change in CME in healthy participants (Stinear and Hornby, 2005; Mrachacz-Kersting et al., 2007; Roy et al., 2007; Jayaram and Stinear, 2008; Kumpulainen et al., 2012, 2015; Mrachacz-Kersting and Stevenson, 2017). Few studies compared PAS to a sham intervention within the same experimental design (Stinear and Hornby, 2005; Mrachacz-Kersting et al., 2007). However, several reported separate experiments where individual components of the intervention were evaluated in isolation (Stinear and Hornby, 2005; Mrachacz-Kersting et al., 2007; Jayaram and Stinear, 2008). Although these experiments support the argument that it is the pairing of the two stimuli in PAS that induces the effect, powered randomized controlled studies with a sham arm are required to rigorously evaluate this. Modulation of upper limb CME induced by PAS suggests a mechanism of action that is cortical in origin (Stefan et al., 2000; Ridding and Uy, 2003). Given that the lower limb is more influenced by spinal input compared to the upper limb (Brouwer and Ashby, 1992; Aymard et al., 2000; Dalpozzo et al., 2002) it could be postulated that spinal mechanisms may contribute to changes in CME following lower limb PAS. However, several small lower limb PAS experiments indicated that spinal excitability measurements (H-reflex and F-waves) remained unchanged following PAS (Mrachacz-Kersting et al., 2007; Roy et al., 2007; Kumpulainen et al., 2012, 2015; Mrachacz-Kersting and Stevenson, 2017).

Some experiments supported the notion that the duration of effect may extend up to an hour following the intervention (Roy et al., 2007), although the magnitude of the effect, with respect to the post-intervention time-point, varied across studies (Stinear and Hornby, 2005; Mrachacz-Kersting et al., 2007; Roy et al., 2007; Jayaram and Stinear, 2008; Kumpulainen et al., 2012, 2015; Mrachacz-Kersting and Stevenson, 2017). Studies of both upper limb PAS, and other non-invasive neuromodulatory interventions, report changes in CME for up to 120 min (Batsikadze et al., 2013; Wischnewski and Schutter, 2016). However, within lower limb PAS literature, the neuromodulatory effect has not been investigated beyond 60 min post-intervention. In a number of experiments included within this review, despite measuring CME at various post-intervention time-points, the authors only analyzed and reported the time-point at which maximum facilitation occurred (Prior and Stinear, 2006; Jayaram and Stinear, 2009), or grouped the results across time-points (Stinear and Hornby, 2005; Jayaram and Stinear, 2009). In these instances, it is not possible to interpret the time-course of the effect.

### Intervention Efficacy of Lower Limb PAS in People With Stroke

This review identified a small number of studies investigating lower limb PAS interventions in people with stroke (Uy et al., 2003; Jayaram and Stinear, 2008, 2009; Rogers et al., 2011). No studies explored the effect of a single session of facilitatory lower limb PAS in people with stroke. One study (Uy et al., 2003) investigated the cumulative effects of 20 facilitatory PAS sessions delivered over 4 weeks; they evaluated CME, muscle strength, range of movement, and gait measurements, in nine people with chronic stroke. Measurements of CME and muscle strength remained unchanged, but there were statistically-significant group improvements in a number of gait parameters (cadence, stride length, and heel-strike). However, given the poor quality of this study, its small sample size, and the absence of a control group, these results may be due to a practice effect and so cautious interpretation is advised.

Several authors assessed single-session inhibitory lower limb PAS interventions in people with stroke, to ascertain whether applying inhibitory PAS to the contralesional M1 and the unaffected lower limb would improve the balance of reciprocal interhemispheric inhibition (Jayaram and Stinear, 2008, 2009; Rogers et al., 2011). This model of rebalancing interhemispheric inhibition has been proposed as an important factor in post-stroke motor recovery (Murase et al., 2004), and therefore inhibiting contralesional M1 activity has been widely adopted in other neuromodulatory interventions (Nowak et al., 2009). Jayaram and Stinear applied inhibitory PAS to the contralesional hemisphere and demonstrated small increases in excitability of the corticospinal pathway to the paretic TA, lasting up to 20 min Jayaram and Stinear (2008, 2009). In contrast when a similar inhibitory PAS intervention was applied to the VM, outcomes were highly variable and there were no significant facilitatory effects in the ipsilesional corticospinal pathway (Rogers et al., 2011). Higher ipsilesional motor thresholds are often observed in people with stroke and MEP latencies can be prolonged due to damaged corticospinal tracts (Koski et al., 2007; Wheaton et al., 2009; Cacchio et al., 2011). It has been asserted that this can cause difficulty estimating the ISI required for applying facilitatory PAS to the ipsilesional hemisphere, making application of inhibitory PAS to the contralesional hemisphere more feasible (Jayaram and Stinear, 2009). However, evidence from other non-invasive neuromodulatory interventions in people with stroke has shown mixed responses to inhibitory interventions applied to the contralesional M1 (Klomjai et al., 2015; Boddington and Reynolds, 2017). The relevance of interhemispheric inhibition to lower limb movement is unclear (Volz et al., 2015; Charalambous et al., 2016) and its role in stroke recovery has been called into question in a recent meta-analysis, which showed insufficient evidence for interhemispheric imbalance after stroke (McDonnell and Stinear, 2017). This suggests that despite methodological challenges, future work should focus on facilitatory interventions delivered to ipsilesional M1.

### Stimulation Parameters

Numerous stimulation parameter settings were employed across the body of research, often in a non-systematic way, making it challenging to unpack how various stimulation parameters may influence the modulation of CME. Based on the depth and quality of the research, it is feasible to comment on the following parameters: ISI, TMS stimulation intensity, TMS coil type, TMS waveform, and the dose of treatment (number of stimulation parings and duration).

#### Dose: Number of Stimulation Pairings and Intervention Duration

Whilst there was a tendency for interventions with more stimulation pairings and longer durations to yield greater changes in CME in healthy people (Mrachacz-Kersting et al., 2007; Roy et al., 2007; Jayaram and Stinear, 2009; Kumpulainen et al., 2012, 2015), the research was not systematic in nature, and methodological differences across studies made direct comparisons challenging. One poor-quality study reported a contrasting finding, demonstrating that a relatively short 5 min intervention with only 60 pairings, increased CME (Roy et al., 2007). At this stage it is not possible to assert the optimal number of pairings or intervention duration, and further research is required.

#### Interstimulus Interval (ISI)

In facilitatory PAS interventions increases in CME were observed with a range of ISIs ranging from 33.5 to 56 ms for TA (Stinear and Hornby, 2005; Prior and Stinear, 2006; Jayaram et al., 2007; Mrachacz-Kersting et al., 2007; Mrachacz-Kersting and Stevenson, 2017) and 48–52ms for SOL (Kumpulainen et al., 2012, 2015). Taking into account an average conduction time of 42–47 ms from the peripheral nerve to the somatosensory cortex, plus a 10 ms central processing delay (Nielsen et al., 1997; Mrachacz-Kersting et al., 2007), these ISI values result in the afferent volley arriving at M1 between 4 ms before, and 23.5 ms after, the TMS stimulation. This is a much larger window (27.5 ms) than reported in upper limb facilitatory PAS interventions, where increases in CME are observed when the afferent volley arrives at the M1 either synchronously with, or just before, the TMS stimulation, within a narrower window (6 ms; Stefan et al., 2000; Carson and Kennedy, 2013). That said, in the lower limb PAS literature, the largest increases in CME were observed when the afferent stimulus and TMS arrived at the M1 at approximately the same time (Mrachacz-Kersting et al., 2007). Substantial CME modulation was also observed in two moderate-quality studies when the ISI was individualized to each participants SEP latency plus a central processing delay (Mrachacz-Kersting et al., 2007; Mrachacz-Kersting and Stevenson, 2017). Although the magnitude of effect still varied across participants, these experiments demonstrated increases in CME in all participants. This contrasts with the upper limb literature which demonstrates conflicting results when ISIs were individualized based on either SEP latency alone (Ilić et al., 2011), or SEP latency plus a central processing (Müller-Dahlhaus et al., 2008; Ilić et al., 2011). It is not clear whether these discrepancies between upper and lower limb PAS interventions are due to differences in interpretation of SEP latency, failure to account for central processing time (Carson and Kennedy, 2013), differences in connectivity between the sensory and motor cortices for the upper and lower limbs (Lotze et al., 2000; Hlustík et al., 2001; Miyai et al., 2001; Luft et al., 2002) or insufficient high quality evidence.

When inhibition of CME was the goal, the moderate-quality research supports using a shorter ISI (18–24 ms; Stinear and Hornby, 2005; Jayaram and Stinear, 2008, 2009; Rogers et al., 2011; Kumpulainen et al., 2015). These ISIs equate to the afferent volley arriving in the M1 28–39 ms after the TMS stimulus, assuming a conduction time of 52–57 ms. In contrast, one study used an ISI in this range, and reported an increase, rather than decrease, in CME; although this poor-quality study also reported facilitatory effects with ISIs of −40, 0, and 35 ms (Roy et al., 2007), which contrasts with the other literature.

With ISIs in the middle range (34–40 ms), the effects of PAS were more inconsistent, with moderate-quality experiments showing either excitation (Stinear and Hornby, 2005; Jayaram et al., 2007), or inhibition (Mrachacz-Kersting et al., 2007; Kumpulainen et al., 2012) and to poor-quality experiments demonstrated a mixture of excitation and inhibition across participants (Uy et al., 2003; Prior and Stinear, 2006). This variability in response may be related to inter-individual variability in conduction times and the influence the task has on conduction-time estimates (Duysens et al., 1990; Brooke et al., 1997). This might suggest that, within this ISI range, it is important to individualize ISIs using SEP latencies. However, as previously suggested, this individualized approach may be more difficult in people with stroke, where altered conduction and central processing times (Koski et al., 2007; Wheaton et al., 2009; Cacchio et al., 2011) can influence the ISI calculation. This may explain why Uy et al. reported excitation in some participants with stroke but no group effect Uy et al. (2003).

Whilst it is asserted that pairing of the afferent volley and the TMS occurs in the M1 (Stefan et al., 2000; Wolters et al., 2003), the definitive timing and location remains unknown. However, the time-dependent nature of PAS resembles STDP observed in single neurons (Markram et al., 1997; Bi and Poo, 1998). Whether through STDP or some other mechanism, the literature reviewed, along with findings from upper limb research (Carson and Kennedy, 2013; Wischnewski and Schutter, 2016; Suppa et al., 2017), indicates that the effects of PAS resemble long-term potentiation (LTP) or long-term depression (LTD) plasticity (Hebb, 1949) with most experiments showing a rapid, change in CME that persists beyond the period of stimulation.

#### TMS Intensity

Research which addressed the effect of the TMS intensity during facilitatory PAS interventions was conflicting. Two moderate-quality experiments (Mrachacz-Kersting et al., 2007; Mrachacz-Kersting and Stevenson, 2017) and one poor-quality experiment (Roy et al., 2007) demonstrated that increases in CME were achieved when both sub- and supra-threshold TMS stimulus intensities were utilized. In contrast, one moderate-quality experiment demonstrated that a very low intensity TMS stimulation was less effective than a higher intensity stimulation (Jayaram et al., 2007); however the intensity of TMS stimulation (100% AMTh during treadmill walking), applied during the seated intervention, was substantially lower than that used in other studies Subthreshold TMS intensities may improve the feasibility of PAS, however further work is required to determine the minimum intensity required to induce changes in CME and to verify whether higher intensity TMS stimulation confers greater benefit.

#### TMS Coil Type

Experiments consistently used either a double-cone or batwing TMS coil, and in those experiments that reported current direction, a posterior-anterior flow was selected. These angular coils were an appropriate choice as they create an electric field which extends to deeper cortical structures (Deng et al., 2013). Coil type is an important consideration, particularly in lower limb PAS where the target M1 area is found deep within the interhemispheric fissure (Terao et al., 1998; Groppa et al., 2012). In addition, a number of other aspects of the TMS delivery method, such as coil location, coil stability and the direction of the induced current may contribute to the efficacy of PAS and therefore should be considered in future research.

#### TMS Pulse Waveform

There is limited evidence with respect to the influence of TMS waveform on CME. One moderate-quality study inferred that the TMS waveform does not influence outcomes, as both monophasic and biphasic waveforms produced similar changes in CME (Mrachacz-Kersting and Stevenson, 2017). However, the authors acknowledged that the findings across the two groups were not directly comparable, as one group evaluated CME with MEPs induced with monophasic TMS, and the other group induced MEPs with biphasic TMS. The potential for biphasic TMS to produce an enhanced effect is supported by work by Kammer et al. (2001) and Sommer et al. (2006), who showed that a biphasic waveform can stimulate neurons positioned in both AP and PA directions, resulting in stimulation of a more diverse collection of neurons, compared to a monophasic waveform. Thus, further research is required to systematically test the effect of different TMS waveforms during PAS in people with stroke.

### Target Muscle State During PAS Interventions

In addition to stimulation parameters, one aspect of the intervention that varied across the studies was the contraction-state of the target muscle. Moderate-quality studies showed that when facilitatory PAS was delivered to the inactive or minimally-active muscle, larger relative increases in CME were observed (Mrachacz-Kersting et al., 2007; Kumpulainen et al., 2012, 2015; Mrachacz-Kersting and Stevenson, 2017) than when delivered during a treadmill walking task (Stinear and Hornby, 2005; Prior and Stinear, 2006; Jayaram et al., 2007; Jayaram and Stinear, 2008, 2009). One possible explanation for this is that during movement there is gating of somatosensory input, such that somatosensory evoked potentials are 30% smaller in walking than standing (Duysens et al., 1990). The low level of PES (120% MTh) utilized in the five studies that delivered PAS during treadmill walking (Stinear and Hornby, 2005; Jayaram et al., 2007; Jayaram and Stinear, 2009) may not have been sufficient to adequately reach the M1, for pairing with TMS stimuli. This idea is supported by one moderate-quality cross-over experiment (Jayaram et al., 2007) where the same 13 participants received facilitatory PAS during (i) sitting, and (ii) walking, but only the sitting intervention resulted in increases in CME.

### Variability in Response to Lower Limb PAS

Across the studies, contrasting results to lower limb PAS were evident within both healthy and stroke populations. Whilst the majority of experiments indicated a significant modulatory effect, several experiments indicated no overall change in CME from baseline (Uy et al., 2003; Prior and Stinear, 2006; Jayaram et al., 2007; Mrachacz-Kersting et al., 2007; Rogers et al., 2011; Kumpulainen et al., 2012) and in some cases modulation was in the opposite direction to what would have been anticipated (Stinear and Hornby, 2005; Prior and Stinear, 2006; Roy et al., 2007; Rogers et al., 2011). Responders and non-responders have been reported in other non-invasive brain stimulation research, whereby the anticipated change in MEP outcomes is reported to occur in only 39–53% of the participants (Hamada et al., 2012; López-Alonso et al., 2014; Wiethoff et al., 2014; Lahr et al., 2016). Non-modifiable factors such as age (Müller-Dahlhaus et al., 2008; Tecchio et al., 2008; Bashir et al., 2014), gender (Cirillo et al., 2010), cranial and cortical anatomy (Hamada et al., 2012, 2014; Wiethoff et al., 2014; McCambridge et al., 2015), genetic profile (Ridding and Ziemann, 2010; Li Voti et al., 2011; Mastroeni et al., 2013; Suppa and Cheeran, 2015), and hormonal fluctuations (Smith et al., 1999; Inghilleri et al., 2004; Sale et al., 2007), and modifiable factors such as time of day (Sale et al., 2008), attention to task (Stefan et al., 2004), use of caffeine, alcohol and nicotine (Specterman et al., 2005; Grundey et al., 2012; Lücke et al., 2014), medications (Ziemann et al., 2015), and motor activity prior, during or after the intervention (Ziemann et al., 2004; Lepage et al., 2012; Goldsworthy et al., 2014; Mang et al., 2014), are all known or hypothesized to influence an individual's response to neuromodulatory interventions. In order to strengthen our understanding of the variability in response to neuromodulatory interventions, it is essential that researchers control for modifiable factors within their study design, while considering non-modifiable factors as co-variates when analyzing and interpreting results.

One moderate-quality study included in this review evaluated the repeatability of the response to PAS in healthy people (Kumpulainen et al., 2012), and demonstrated that whilst there was large inter-individual variability following facilitatory PAS (mean increase 46 ± 52%, *n* = 8), individuals demonstrated a repeatable response to the same PAS intervention delivered 3 days later (mean increase 36 ± 32%, ICC = 0.85). This finding contrasts with upper limb PAS studies, where the individual responses to PAS were highly variable between sessions (Fratello et al., 2006; Sale et al., 2007). Such variability within and between individuals emphasizes the need for caution when extrapolating findings.

### TMS Measurement Considerations

Another explanation for the variability in results is the different methods employed for assessing CME across the studies. In general, PAS experiments which recorded resting MEPs showed larger relative changes in MEP amplitude (Mrachacz-Kersting et al., 2007; Roy et al., 2007; Kumpulainen et al., 2012, 2015; Mrachacz-Kersting and Stevenson, 2017). In contrast, experiments which recorded MEPs during a functional task (treadmill walking) showed smaller relative changes in MEP amplitude (Stinear and Hornby, 2005; Prior and Stinear, 2006; Jayaram et al., 2007; Jayaram and Stinear, 2008, 2009; Rogers et al., 2011). However, it must be considered that during a functional task, small relative changes in MEPs represent larger absolute changes. In addition to recording MEPs in the resting muscle or during functional tasks, five experiments recorded active MEPs during a voluntary contraction (5–50% MVC) (Uy et al., 2003; Roy et al., 2007; Kumpulainen et al., 2012, 2015). Only two of these experiments demonstrated significant changes in active MEP amplitudes (Roy et al., 2007; Kumpulainen et al., 2015), yet four of the five experiments demonstrated changes in resting MEPs (Roy et al., 2007; Kumpulainen et al., 2012, 2015). This suggests that it may be more difficult to induce changes in active MEPs than resting MEPs. A possible explanation may relate to the different neurons being recruited during resting and active MEPs (low threshold vs. high threshold; Hess et al., 1987; Di Lazzaro et al., 2004; Rossini et al., 2015), and the task-specificity of cortical circuitry. For example, when PAS is delivered to an inactive muscle, and changes are seen in resting but not active MEPs (Roy et al., 2007; Kumpulainen et al., 2012), this may be because the changes in the specific circuitry involved were only measurable in the resting condition. However, this idea is contradicted by other results where PAS was delivered to an inactive target muscle and changes occurred in MEPs recorded during functional tasks (Jayaram et al., 2007; Jayaram and Stinear, 2008).

Across the studies included in this review TMS checklist scores indicate moderate methodological quality (mean 66%, SD 9%, range 38–75%). Improvements in the methodological quality of TMS measurement may improve the ability to detect changes in response to the intervention. Whilst we have touched on the importance of reducing intra- and inter-individual variability by controlling for modifiable factors within study designs and addressing non-modifiable factors as co-variates, future research should also consider methods to further minimize trial to trial MEP variability. This variability is believed to be linked to continuous fluctuation in cortical activity rather than noise in the signal (Bergmann et al., 2012). One way to potentially minimize this variability in TMS measurement is by delivering real time electroencephalogram (EEG) triggered TMS. Synchronizing each TMS pulse with specific sensorimotor oscillatory phases in alpha bands of EEG signals enables consistency in the delivery of the stimulus, and in turn reduces variability in trial to trial MEPs (Zrenner et al., 2018; Schaworonkow et al., 2019). This method may provide a more comprehensive understanding of the effect of the intervention on CME.

The use of resting MEPs as an outcome measure likely creates a selection bias by favoring people with stroke with less severe impairment. Future research should aim to investigate a more heterogeneous sample, inclusive of people with moderate to severe disability, which would be more reflective of the population of interest. TMS measurements during a voluntary contraction, or functional activity such as treadmill walking, may be best suited to this patient population. Furthermore, whilst studies which investigate CME provide insight into the neurophysiological effects of PAS, we cannot extrapolate this effect to behavioral changes such as improved motor performance. Therefore, it is essential to consider pertinent behavioral measurement tools that can ascertain the cumulative effects of lower limb PAS on motor performance in patient populations, before considering the translation of lower limb PAS into clinical practice.

### Strengths and Limitations

This is the first systematic review to determine the efficacy of PAS on lower limb CME in healthy and stroke populations. In doing so, the review provides a detailed synthesis of the literature by explicitly considering the influence of stimulation parameter selection and methodological quality of the research.

There are a number of limitations to this review. Firstly, whilst a comprehensive set of search terms were predefined and piloted to ensure maximal retrieval, pertinent literature may have been overlooked. It is possible that publication bias may account for some of the effects reported. Secondly, it was not possible to obtain all outcome data and their variance estimates, requiring changes in CME to be interpreted from graphical representations. These approximations may have resulted in inaccuracy. Thirdly, this review does not include a meta-analysis; until the body of evidence grows in methodological rigor, it is not possible to combine studies statistically. Future robust randomized controlled trials are required to confirm the efficacy of lower limb PAS on CME in healthy and stroke populations, and to determine the influence of stimulation parameter selection.

### Future Recommendations

The studies reviewed provide moderate-to-poor quality evidence that a single session of lower limb PAS can modulate CME in healthy and stroke populations for up to an hour. There is insufficient evidence for the optimal stimulation parameter settings. In order to advance our understanding of the therapeutic value of lower limb PAS interventions in stroke rehabilitation, future research should:

1. Improve methodological rigor by making comparisons with a control group, performing power calculations, reducing selection bias, blinding participants and assessors, and controlling for confounding variables.
2. Take a systematic approach to testing the efficacy of different PAS stimulation parameters: ISI; number of pairings; pairing frequency; TMS intensity and waveform; PES frequency and pulse width; electrode location; and the target muscle state.
3. Consider individualizing ISIs based on SEP latencies, as per evidence from moderate quality studies (Mrachacz-Kersting et al., 2007; Mrachacz-Kersting and Stevenson, 2017).
4. Consider how the muscle contraction state during MEP measurement influences the ability to detect a significant effect, and consider testing MEPs under multiple conditions (resting, active, and functional muscle states).
5. Explicitly report methods to control confounding variables during CME measurement and undertake measurement procedures according to best practice (Chipchase et al., 2012; Rossini et al., 2015).
6. Include behavioral measures (i.e., motor performance) and consider investigating the cumulative effects of PAS in patient populations.
7. Define a statistical analysis plan *a priori* and ensure that all results are presented. Report whether absolute and relative changes in CME are used for statistical analysis. To facilitate the synthesis of studies, raw data should be presented. Clearly identify any secondary exploratory analyses.
8. Measure CME at multiple post-intervention time points but avoid analyzing multiple time points together. This will facilitate our understanding about the time-course of neuromodulatory effects. From a neurorehabilitation perspective, this knowledge will allow researchers and clinicians to consider how PAS might be best integrated with standard rehabilitation to take advantage of the window of excitation.

## Conclusion

This systematic review critically assessed the efficacy of PAS on lower limb CME in healthy and stroke populations, whilst explicitly considering methodological rigor and stimulation parameter selection. Findings from this review suggest lower limb PAS requires further investigation before considering its translation into stroke rehabilitation. There is moderate-to-poor quality evidence to support the efficacy of a single session of lower limb PAS to modulate CME in healthy and stroke populations. Stimulation parameter selection appears to influence efficacy, but robust conclusions are hindered by a lack of high-quality studies that systematically compare different PAS-delivery methods employed across studies. To advance our understanding in the field, we propose eight key recommendations for the design of future research.

## Ethics Statement

Ethical approval and consent were obtained in all the individual studies that were included in this review.

## Author Contributions

GA, DT, and NS conceptualized the study. GA performed the search strategy, extracted study data, and critically revised and wrote the manuscript with contributions from SO. GA and NS independently screened articles for eligibility and independently completed quality check lists for included articles. GA, NS, SO, and DT contributed to the interpretation of the results, read and approved the final manuscript. NS edited the manuscript. NS and DT carried out a critical revision of the manuscript.

### Conflict of Interest Statement

The authors declare that the research was conducted in the absence of any commercial or financial relationships that could be construed as a potential conflict of interest.

## Abbreviations

AMTh: Active motor threshold
CME: Corticomotor excitability
CPN: Common peroneal nerve
D&B QC: Modified Downs and Black Quality Checklist
DF: dorsiflexion
EEG: Electroencephalogram
HZ: Herts
ICF: Intracortical facilitation
ISI: Interstimulus interval
LTD: Long term depression
LTP: Long term potential
M1: Motor cortex
MEP: Motor evoked potential
MEPmax: maximum MEP amplitude
MH: Medial hamstrings
ms: Millisecond
MTh: Motor threshold
mV: Millivolt
MVC: Maximum voluntary contraction
PAS: Paired associative stimulation
PAS+: Facilitatory paired associative stimulation
PAS-: Inhibitory paired associative stimulation
PES: Peripheral electrical stimulus
PL: Peroneous longus
PTh: Perceptual threshold
Rpm: Revolutions per minute
RMTh: Resting motor threshold
rTMS: Repetitive transcranial magnetic stimulation
SEP: Sensory evoked potential
SICI: Short interval intracortical stimulation
SOL: Soleus
STh: Sensory threshold
STDP: Spike timing dependent neural plasticity
TA: Tibialis anterior
TDCS: Transcranial direct current stimulation
TMS: Transcranial magnetic stimulation
TN: Tibial nerve
TMS QC: TMS Quality Checklist
VL: Vastus lateralis
VM: Vastus medialis.
