# Supplementary material for: A Systematic Review of Paired Associative Stimulation (PAS) to Modulate Lower Limb Corticomotor Excitability: Implications for Stimulation Parameter Selection and Experimental Design
Source: Front Neurosci. 2019 Aug 27;13:895. doi: 10.3389/fnins.2019.00895 (PMC6718871; doi:10.3389/fnins.2019.00895)
Supplement: Supplementary file 2 [file Table_2.DOCX]

**Supplementary material: Table 2. TMS checklist scores** (Chipchase 2012)

|  | **Healthy Studies** | | | | | | | | | | | | | | | | **Stroke Studies** | | | | | | | | |
| --- | --- | --- | --- | --- | --- | --- | --- | --- | --- | --- | --- | --- | --- | --- | --- | --- | --- | --- | --- | --- | --- | --- | --- | --- | --- |
|  | Stinear  (2005) | | Prior  (2006) | | Jayaram  (2007) | | Mrachaz-Kresting (2007) | | Roy (2007) | | Kumpula-inen (2012) | | Kumpula-inen (2015) | | Mrachaz-Kresting (2017) | | Uy  (2003) | | Jayaram (2008) | | Jayaram  (2009) | | Rogers (2011) | | |
|  | R | C | R | C | R | C | R | C | R | C | R | C | R | C | R | C | R | C | R | C | R | C | R | C |  |
| ***Participant factors*** |  |  |  |  |  |  |  |  |  |  |  |  |  |  |  |  |  |  |  |  |  |  |  |  |  |
| Age of participants | 1 | 1 | 1 | 1 | 1 | 1 | 1 | 0 | 1 | 0 | 1 | 1 | 1 | 1 | 1 | 1 | 1 | 0 | 1 | 1 | 1 | 1 | 1 | 1 |  |
| Gender of participants | 1 | NA | 1 | NA | 1 | NA | 1 | NA | 1 | NA | 1 | NA | 1 | NA | 1 | NA | 1 | NA | 1 | NA | 1 | NA | 1 | NA |  |
| Handedness / Footedness of participants | 1 | 1 | 0 | 0 | 0 | 0 | 0 | 0 | 0 | 0 | 0 | 0 | 0 | 0 | 1 | 1 | 0 | 0 | 1 | 1 | 0 | 0 | 0 | 0 |  |
| Participants prescribed medication | 0 | 0 | 0 | 0 | 0 | 0 | 0 | 0 | 0 | 0 | 0 | 0 | 0 | 0 | 0 | 0 | 0 | 0 | 0 | 0 | 0 | 0 | 0 | 0 |  |
| Use of CNS active drugs | 0 | 0 | 0 | 0 | 0 | 0 | 0 | 0 | 0 | 0 | 0 | 0 | 0 | 0 | 0 | 0 | 0 | 0 | 0 | 0 | 0 | 0 | 0 | 0 |  |
| Presence of neurological/psychiatric disorders in healthy participants | 1 | 1 | 1 | 1 | 1 | 1 | 1 | 1 | 0 | 0 | 0 | 0 | 1 | 1 | 1 | 1 | NA | NA | 1 | 1 | NA | NA | 1 | 1 |  |
| Any medical conditions | 0 | 0 | 0 | 0 | 0 | 0 | 0 | 0 | 0 | 0 | 0 | 0 | 1 | 0 | 0 | 0 | 0 | 0 | 0 | 0 | 0 | 0 | 0 | 0 |  |
| History of specific repetitive motor activity | 0 | 0 | 0 | 0 | 0 | 0 | 0 | 0 | 0 | 0 | 0 | 0 | 1 | 1 | 0 | 0 | 0 | 0 | 0 | 0 | 0 | 0 | 0 | 0 |  |
| ***Methodological factors*** |  |  |  |  |  |  |  |  |  |  |  |  |  |  |  |  |  |  |  |  |  |  |  |  |  |
| Position and contact of EMG electrodes | 0 | 0 | 1 | 1 | 1 | 0 | 1 | 1 | 1 | 1 | 1 | 1 | 1 | 1 | 1 | 1 | 0 | 0 | 1 | 0 | 1 | 0 | 1 | 1 |  |
| Amount of relaxation/contraction of target muscles | 1 | 1 | 1 | 1 | 1 | 1 | 1 | 1 | 1 | 1 | 1 | 1 | 1 | 1 | 1 | 1 | 1 | 1 | 1 | 1 | 1 | 1 | 1 | 1 |  |
| Prior motor activity of the muscles to be tested | 1 | 1 | 1 | 1 | 1 | 1 | 0 | 0 | 1 | 1 | 0 | 0 | 0 | 0 | 0 | 0 | 0 | 0 | 1 | 1 | 1 | 1 | 1 | 1 |  |
| Level of relaxation of muscles other than those being tested | NA | 1 | NA | 1 | NA | 0 | NA | 1 | NA | 1 | NA | 1 | NA | 0 | NA | 0 | NA | 0 | NA | 0 | NA | 1 | NA | 0 |  |
| Coil type (size and geometry) | 1 | 1 | 1 | 1 | 1 | 1 | 1 | 1 | 1 | 1 | 1 | 1 | 1 | 1 | 1 | 1 | 1 | 1 | 1 | 1 | 1 | 1 | 1 | 1 |  |
| Coil orientation | 1 | 1 | 1 | 1 | 1 | 1 | 1 | 1 | 1 | 1 | 0 | 0 | 0 | 0 | 1 | 1 | 0 | 0 | 1 | 1 | 1 | 1 | 1 | 1 |  |
| Direction of induced current in the brain | 0 | 0 | 1 | 1 | 1 | 1 | 1 | 1 | 1 | 1 | 0 | 0 | 0 | 0 | 1 | 1 | 0 | 0 | 1 | 1 | 1 | 1 | 1 | 1 |  |
| Coil location and stability (with or without neuro-navigation system) | 1 | 1 | 1 | 1 | 1 | 1 | 1 | 1 | 1 | 1 | 1 | 1 | 1 | 1 | 0 | 0 | 0 | 0 | 1 | 1 | 1 | 1 | 1 | 1 |  |
| Type of stimulator used (e.g. brand) | 1 | 1 | 1 | 1 | 1 | 1 | 1 | 1 | 1 | 1 | 1 | 1 | 1 | 1 | 1 | 1 | 1 | 1 | 1 | 1 | 1 | 1 | 1 | 1 |  |
| Stimulation intensity | 1 | 1 | 1 | 1 | 1 | 1 | 1 | 1 | 1 |  | 1 | 1 | 1 | 1 | 1 | 1 | 1 | 1 | 1 | 1 | 1 | 1 | 1 | 1 |  |
| Pulse shape (monophasic or biphasic) | 0 | 0 | 0 | 0 | 0 | 0 | 0 | 0 | 0 | 0 | 1 | 1 | 1 | 1 | 1 | 1 | 0 | 0 | 0 | 0 | 0 | 0 | 0 | 0 |  |
| Determination of optimal hotspot | 1 | 0 | 0 | 0 | 0 | 0 | 1 | 1 | 1 | 1 | 1 | 1 | 0 | 1 | 1 | 1 | 1 | 1 | 1 | 1 | 1 | 1 | 0 | 0 |  |
| The time between MEP trials | 1 | 1 | 1 | 1 | 1 | 1 | 1 | 1 | 1 | 1 | 0 | 0 | 0 | 0 | 1 | 1 | 0 | 0 | 1 | 1 | 1 | 1 | 1 | 1 |  |
| Time between days of testing | 1 | 1 | 1 | 1 | 1 | 1 | 1 | 1 | 1 | 1 | 1 | 1 | NA | NA | 1 | 1 | 1 | 1 | NA | NA | 1 | 1 | NA | NA |  |
| Subject attention (level of arousal) during testing | 1 | 1 | 0 | 0 | 1 | 1 | 1 | 1 | 0 | 0 | 1 | 1 | 1 | 1 | 1 | 1 | 0 | 0 | 1 | 1 | 1 | 1 | 0 | 0 |  |
| Method of determining threshold (active/resting) | 1 | 1 | 1 | 1 | 1 | 1 | 1 | 1 | 1 | 1 | 1 | 1 | 1 | 1 | 1 | 1 | 0 | 0 | 1 | 1 | 1 | 1 | 1 | 1 |  |
| Number of MEP measures made | 1 | 1 | 1 | 1 | 1 | 1 | 1 | 1 | 1 | 1 | 1 | 1 | 1 | 1 | 1 | 1 | 1 | 1 | 1 | 1 | 1 | 1 | 1 | 1 |  |
| ***Paired pulse only*** |  |  |  |  |  |  |  |  |  |  |  |  |  |  |  |  |  |  |  |  |  |  |  |  |  |
| Intensity of test pulse | NA | NA | NA | NA | NA | NA | NA | NA | 1 | 1 | NA | NA | NA | NA | NA | NA | NA | NA | NA | NA | NA | NA | NA | NA |  |
| Intensity of conditioning pulse | NA | NA | NA | NA | NA | NA | NA | NA | 1 | 1 | NA | NA | NA | NA | NA | NA | NA | NA | NA | NA | NA | NA | NA | NA |  |
| Inter-stimulus interval | NA | NA | NA | NA | NA | NA | NA | NA | 1 | 1 | NA | NA | NA | NA | NA | NA | NA | NA | NA | NA | NA | NA | NA | NA |  |
| ***Analytical factors*** |  |  |  |  |  |  |  |  |  |  |  |  |  |  |  |  |  |  |  |  |  |  |  |  |  |
| Method of determining MEP size during analysis | 1 | 1 | 1 | 1 | 1 | 1 | 1 | 1 | 1 | 1 | 1 | 1 | 1 | 1 | 1 | 1 | 1 | 1 | 1 | 1 | 1 | 1 | 1 | 1 |  |
| Size of unconditioned MEP | NA | NA | NA | NA | NA | NA | NA | NA | 1 | 1 | NA | NA | NA | NA | NA | NA | NA | NA | NA | NA | NA | NA | NA | NA |  |
| ***Totals*** | 18 | 17 | 17 | 17 | 18 | 16 | 18 | 17 | 21 | 19 | 15 | 15 | 16 | 15 | 19 | 18 | 10 | 8 | 19 | 17 | 18 | 17 | 16 | 15 |  |
| % Score | 72 | 68 | 68 | 68 | 72 | 64 | 72 | 68 | 72 | 66 | 60 | 60 | 67 | 63 | 76 | 72 | 42 | 33 | 79 | 71 | 75 | 71 | 67 | 63 |  |
| **Overall % Score** | **70** | | **68** | | **68** | | **70** | | **69** | | **60** | | **65** | | **74** | | **38** | | **75** | | **73** | | **65** | | |

R= reported, C= controlled, 1= yes, 0= no, NA= non applicable.
